# Evaluation of Intracranial Hypertension in Patients With Hypertensive Intracerebral Hemorrhage Using Texture Analysis

**DOI:** 10.3389/fneur.2022.832234

**Published:** 2022-03-16

**Authors:** Yingchi Shan, Yihua Li, Xiang Wu, Jiaqi Liu, Guoqing Zhang, Yajun Xue, Guoyi Gao

**Affiliations:** ^1^Department of Neurosurgery, Shanghai General Hospital, Shanghai Jiao Tong University School of Medicine, Shanghai, China; ^2^Department of Neurosurgery, The People's Hospital of Qiannan, Guizhou, China

**Keywords:** hypertensive intracerebral hemorrhage, computed tomography, texture analysis, noninvasive evaluation, intracranial hypertension

## Abstract

**Purpose:**

Texture analysis based on clinical images had been widely used in neurological diseases. This study aimed to achieve depth information of computed tomography (CT) images by texture analysis and to establish a model for noninvasive evaluation of intracranial pressure (ICP) in patients with hypertensive intracerebral hemorrhage (HICH).

**Methods:**

Forty-seven patients with HICH were selected. Related CT images and ICP value were collected. The morphological features of hematoma volume, midline shift, and ventriculocranial ratio were measured. Forty textural features were extracted from regions of interest. Four models were established to predict intracranial hypertension with morphological features, textural features of anterior horn, textural features of temporal lobe, and textural features of posterior horn.

**Results:**

Model of posterior horn had the highest ability to predict intracranial hypertension (AUC = 0.90, F1 score = 0.72), followed by model of anterior horn (AUC = 0.70, F1 score = 0.53) and model of temporal lobe (AUC = 0.70, F1 score = 0.58), and model of morphological features displayed the worst performance (AUC = 0.42, F1 score = 0.38).

**Conclusion:**

Texture analysis can realize interpretation of CT images in depth, which has great potential in noninvasive evaluation of intracranial hypertension.

## Introduction

Hypertensive intracerebral hemorrhage (HICH) is one of the most common critical diseases in neurosurgery, with high morbidity, disability, mortality, and recurrence rates (1–3). The increase of intracranial pressure (ICP) after intracerebral hemorrhage directly causes the decrease of cerebral perfusion pressure, and finally leads to cerebral ischemia, hypoxia, and even cerebral hernia (4). Timely and efficient monitoring of ICP plays an important role in identifying intracranial physiological and pathological changes (5). In recent years, various methods of noninvasive ICP monitoring have been used in clinical practice, with the characteristics of low cost, wide range of use and fewer complications (6–8). Computed tomography (CT) image, as one of the most commonly used clinical examination, plays an essential role in the evaluation of patients' condition (9). In addition, relatively perfect scoring scales and guidelines have been formed for the morphological analysis of CT images, which can be used for the evaluation of ICP to a certain extent, but it largely depends on the clinical experiences of doctors and is difficult to be accurate and quantitative (10, 11). This study aims to analyze nonvisual features of CT images by texture analysis, with machine learning being used to capture ICP signals, and then the performance of predicting ICP by CT can be improved.

## Materials and Methods

### Study Design and Setting

Clinical data of patients with HICH admitted to the Department of Neurosurgery, Shanghai General Hospital from January 2019 to December 2020 were collected and analyzed. Inclusion criteria were as follows: (1) patients admitted to emergency department for spontaneous intracranial hemorrhage, with a history of hypertension; (2) with the main lesion of intracerebral hematoma; (3) received invasive ICP monitoring according to the guidelines (12) for the management of HICH; (4) received emergency cranial CT scan within 60 min of ICP monitoring. Exclusion criteria were as follows: (1) patients with a history of traumatic brain injury, cerebral infarction, brain tumor, or other neurological diseases or cranial surgical interventions that might result in an abnormal anatomical structure; (2) with previous coagulopathy and blood system related diseases; (3) a large amount of subarachnoid hemorrhage or ventricular hemorrhage being showed on CT images, with aneurysms, moyamoya disease, vascular malformations, or other vascular anatomical abnormalities detected by cranial CTA or angiography in follow-up treatment. The study protocol conformed to the ethical guidelines of the Declaration of Helsinki, and this study was approved by the Ethics Committee of Shanghai General Hospital, Shanghai Jiao Tong University School of Medicine. Participants' right to know was fully guaranteed and indicated in the ethical approval document.

### Data Sources and Measurements

In addition to the baseline characteristics, we collected and analyzed the last cranial CT before the surgery and the initial ICP value immediately after ICP sensor (Integra, USA) insertion. In this study, the ICP sensor was inserted into the ventricle before performing a craniotomy for hematoma removal, and the location of the sensor was verified by cranial CT after surgery. The CT related features were acquired from the Digital Imaging and Communications in Medicine (DICOM) file of the last cranial CT before ICP monitoring using a 64-slice spiral CT machine (General Electric Medical Systems, USA). As per the routine protocol of a CT scan, the CT slices were parallel to the orbitomeatal plane from the foramen magnum to the vertex. The scanning slice thickness was 1 mm.

In this study, hematoma volume, midline shift, and ventriculocranial ratio (VCR) were included in the morphological features, and the final results were the average of the measurements by two clinical experts. The specific measuring method is as follows: (1) the DICOM files of CT were imported into 3D slicer software, using the Threshold function under the Editor module to complete the 3D reconstruction of the hematoma and obtain the hematoma volume; (2) the slice with maximum midline shift was selected, and the perpendicular distance between the septum pellucidum and cranial midline (median ridge of the fronto-parietal bone) was measured at the level of Monro's foramen; (3) the lateral ventricle width between the heads of the caudate nuclei and the width of the same horizontal cranial cavity were measured at the slice which was 5 cm above the orbitomeatal plane; ratio of the two width was VCR. These three morphological features were then included into random forest model to evaluate the probability of intracranial hypertension (ICP ≥ 20 mmHg) (12) as the morphological features related model (MF model).

**Figure 1:**
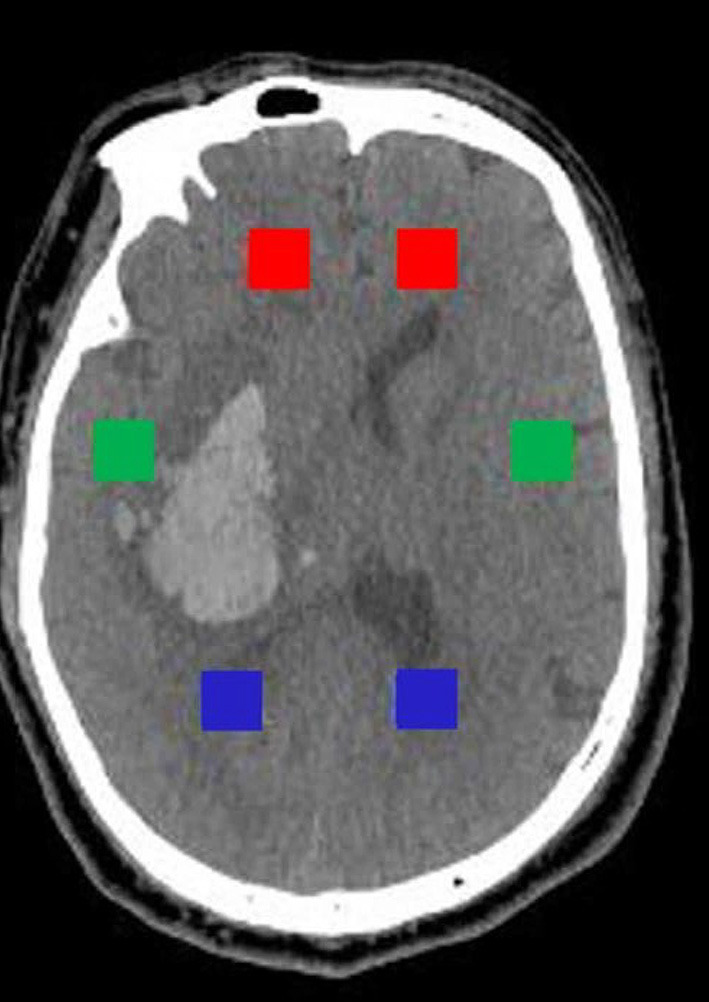
Selection of six rectangular ROI with size of 20 pixels × 20 pixels. The red, green, and blue rectangles, respectively, represent ROI of the anterior horn, the temporal lobe, and the posterior horn.


The slice with upper part of the third ventricle (about 6 cm above the orbitomeatal plane) was selected for the extraction of textural features. “Pydicom” module in Python 3.8 was used to extract six rectangular regions of interest (ROI) with size of 20 pixels × 20 pixels from the bilateral anterior horn of lateral ventricle, bilateral temporal lobe and bilateral posterior horn of lateral ventricles (the bilateral ROI is symmetrical as shown in Figure 1). On the one hand, the ROIs were selected according to CT values (the threshold of CT value was set to ≤50 HU and ≥0 HU, HU was short for Hounsfield Unit); on the other hand, manual verification was carried out to avoid ROIs falling into the hematoma or the ventricle. “Radiomics” module was used to extract a total of 40 textural features including gray level cooccurrence matrix (GLCM, 24 features) and gray level run length matrix (GLRLM, 16 features) from six ROI, respectively. Next, there were three steps for processing textural features. Firstly, the value of features from six ROI were transformed into variation rate related to three areas (anterior horn of lateral ventricle, temporal lobe, and posterior horn of lateral ventricle). The formula was as follows:

$$\begin{array}{l} R=|\frac{F_{Hemorrhagic\;lateral}-F_{Contralateral}}{F_{Hemorrhagic\;lateral}}| \end{array}$$

Secondly, the variation rate of three groups were standardized. The formula was as follows:


$$\begin{array}{l} R_{new}=\frac{R-R_{\min}}{R_{\max}-R_{\min}} \end{array}$$

**Figure 2:**
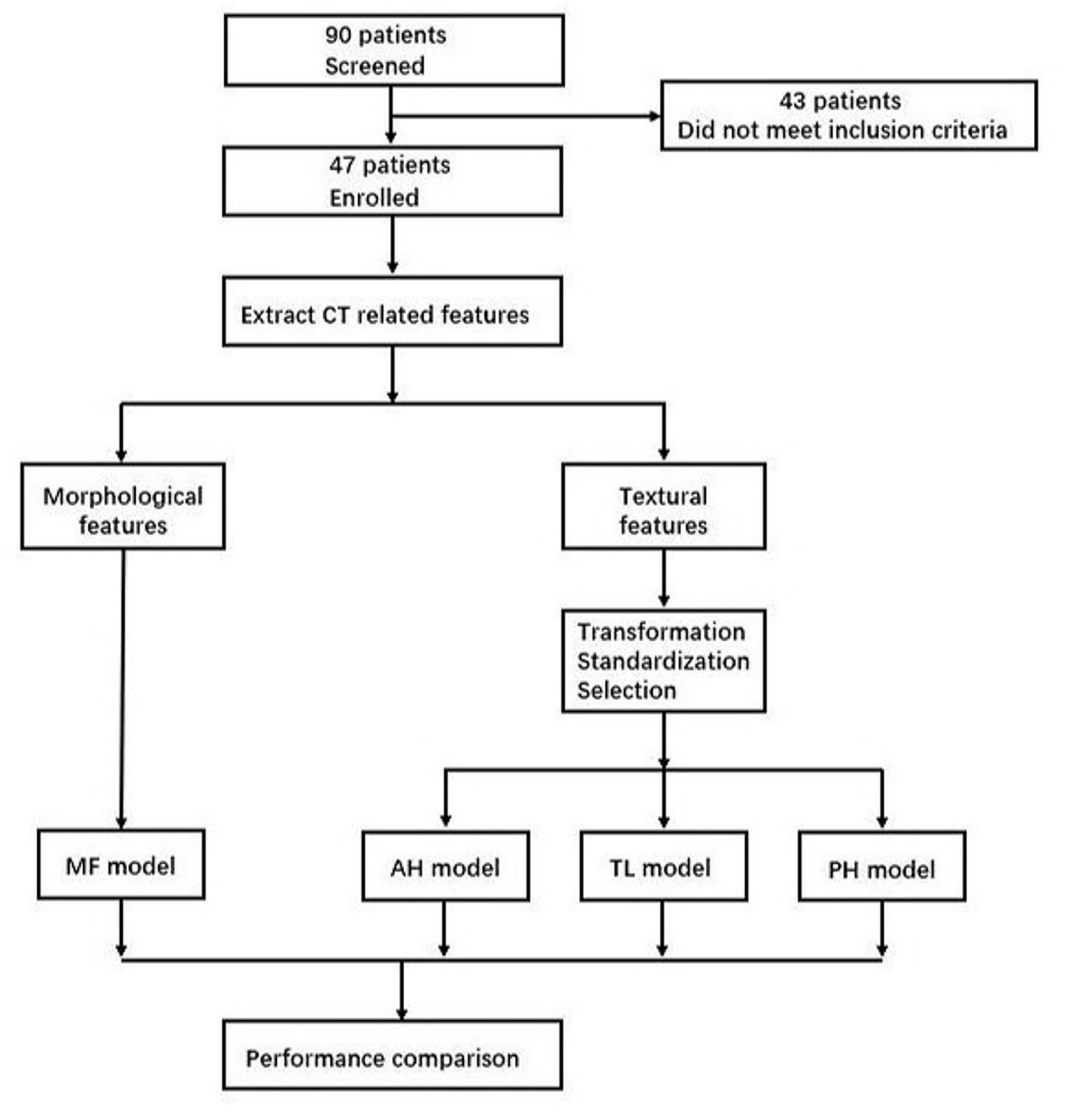
Model establishment and analysis process. MF, morphological features; AH, anterior horn; TL, temporal lobe; PH, posterior horn.

Thirdly, recursive feature elimination method (RFE) was used for feature selection in three groups, respectively. The importance of each feature was obtained by random forest classifier, the least important feature was removed from the current feature set, and this step was repeated on the feature set. To avoid overfitting, cross validation (RFECV) was used to find the optimal number of features. According to the features selected for three groups, three random forest models were established to evaluate the probability of intracranial hypertension (ICP ≥ 20 mmHg), including anterior horn model (AH model), temporal lobe model (TL model), and posterior horn (PH model). The establishment and analysis process of above models are shown in Figure 2.

### Statistical Analysis

Python 3.8 was used for statistical analysis of the data. Numpy module and Pandas module were used for data operation and sorting, RFECV module of Sklearn was used for feature selection, RandomForestClassifier module of Sklearn was used for model establishment, and Matplotlib module is used for drawing. Continuous variables subject to normal distribution were expressed as the mean (M) ± standard deviation (SD), continuous variables not subject to normal distribution were expressed as the median and interquartile range (IQR), and categorical variables were expressed as the frequency and percentage. Accuracy, precision, recall, and F1 score were used to evaluate the performance of each model. The area under the receiver operator characteristic (ROC) curve was used in all four models to assess discrimination.

## Results

As shown in Table 1, a total of 47 patients with HICH were included in this study, among which 30 were males (63.83%) and 17 were females (36.17%). Bleeding sites include basal ganglia (35, 74.47%), thalamus (5, 10.64%) and cerebral cortex (7, 14.89%), and six cases (12.77%) were accompanied by a small amount of subarachnoid hemorrhage. The median Glasgow Coma Score (GCS) was 6 (IQR: 4–7) at the time of emergency admission. Median systolic pressure was 146 mmHg (IQR: 135–157) upon arrival. The median ICP of all patients was 25 mmHg (IQR: 15–30), and 32 patients (68.09%) were diagnosed with intracranial hypertension. Median hematoma volume was 52.40 mL (IQR: 37.25–78.94 ml), median midline shift was 6.61 mm (IQR: 3.95–9.21 mm), and median ventriculocranial ratio was 0.24 (IQR: 0.16–0.30).

**Table 1:** Initial ICP and morphological features of patients.

	**Patients with HICH** **(*n* = 47)**
Systolic pressure (mmHg)	146 (135–157)
GCS score	6 (4–7)
Initial ICP (mmHg)	25 (15–30)
>20 mmHg	32 (68.09%)
Location of hemorrage
*Thalamus*	5 (10.64%)
*Basal ganglia*	35 (74.47%)
*Cerebral cortex*	7 (14.89%)
With subarachnoid hemorrhage	6 (12.77%)
Hematoma volume (ml)	52.40 (37.25–78.94)
Midline shift (mm)	6.61 (3.95–9.21)
Ventriculocranial ratio	0.24 (0.16–0.30)

The selected textural features and variation rate of anterior horn, temporal lobe, and posterior horn are shown in Table 2. After RFECV selection, a total of 12 features were extracted from the anterior horn of the lateral ventricle, and the cross-validation score was the highest at this time. As above, 10 features were extracted from temporal lobe and 12 features were extracted from posterior horn of lateral ventricle. The illustration of all features extracted was added in the Supplementary Material.

**Table 2:** Selected features and their variation rate.

**Selected features**	**Variation rate**
Anterior horn (12 features)
Autocorrelation	0.09 (0.05~0.16)
Cluster shade	0.71 (0.46~1.66)
Idm	0.05 (0.03~0.07)
Joint average	0.05 (0.02~0.09)
Joint energy	0.27 (0.16~0.40)
Maximum probability	0.16 (0.10~0.24)
Sum average	0.05 (0.02~0.09)
Gray level variance	0.47 (0.24~0.75)
High gray level run emphasis	0.59 (0.32~0.79)
Run length nonuniformity	0.22 (0.10~0.34)
Short run emphasis	0.20 (0.09~0.45)
Short run low gray level emphasis	0.25 (0.13~0.39)
Temporal lobe (10 features)
Cluster prominence	0.65 (0.32~0.91)
Id	0.05 (0.02~0.08)
Idm	0.05 (0.02~0.08)
Joint average	0.06 (0.03~0.12)
Maximum probability	0.11 (0.06~0.23)
Gray level nonuniformity	0.12 (0.05~0.23)
High gray level run emphasis	0.22 (0.13~0.38)
Long run high gray level emphasis	0.49 (0.22~0.85)
Run length nonuniformity	0.26 (0.14~0.52)
Run percentage	0.22 (0.09~0.40)
**Posterior horn (12 features)**
Cluster prominence	0.40 (0.23~0.88)
Cluster shade	0.82 (0.34~2.37)
Correlation	0.92 (0.57~1.75)
Imc1	0.45 (0.25~0.79)
Imc2	0.32 (0.14~0.56)
MCC	0.42 (0.15~0.60)
Sum squares	0.33 (0.13~0.73)
Long run emphasis	0.26 (0.16~0.45)
Run entropy	0.15 (0.09~0.23)
Run length nonuniformity normalized	0.34 (0.18~0.55)
Run percentage	0.12 (0.07~0.23)
Short run low gray level emphasis	0.17 (0.07~0.26)

**Figure 3:**
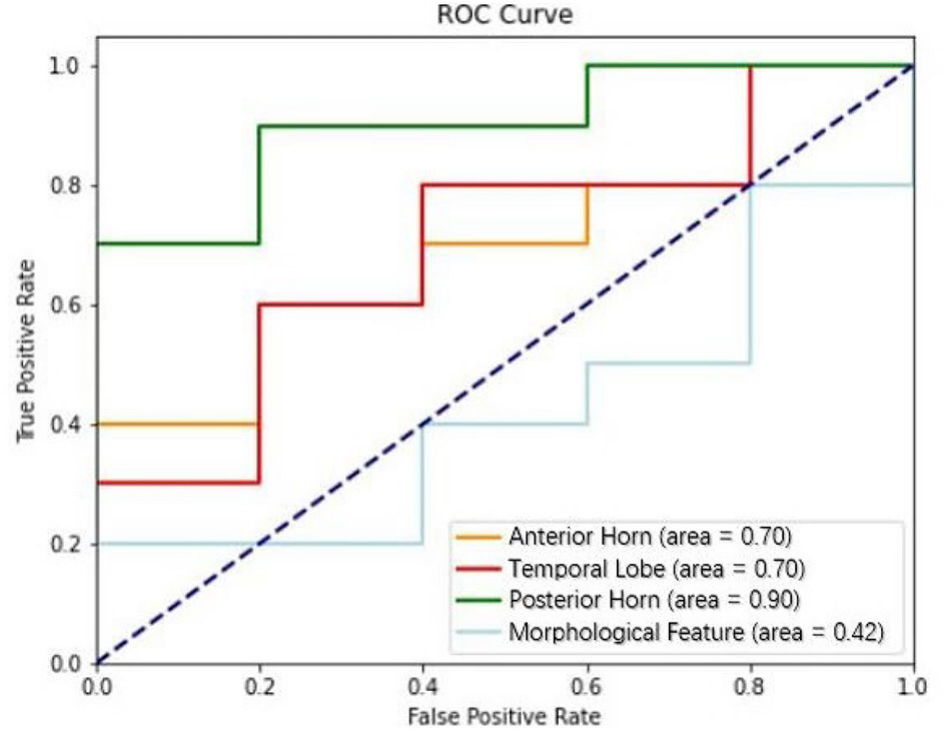
Performance of models based on textural features (anterior horn, temporal lobe, and posterior horn) and morphological features in discriminating intracranial hypertension; the AUCs for these models were 0.70, 0.70, 0.90, and 0.42, respectively.

The performance of the MF, AH, TL, and PH models in predicting intracranial hypertension is shown in Table 3 and Figure 3. PH model had the strongest prediction ability of intracranial hypertension, TL model and AH model had the same prediction ability and were inferior to PH model, and MF model had the worst prediction ability of intracranial hypertension.

**Table 3:** Performance of models based on textural features and morphological features.

	**Textural** **features** **(anterior horn)**	**Textural** **features** **(temporal lobe)**	**Textural** **features** **(posterior horn)**	**Morphological** **features**
Accuracy	66.67%	73.33%	80.00%	60.00%
Precision	59.62%	85.71%	88.46%	32.14%
Recall	55.00%	60.00%	70.00%	45.00%
F1 score	0.53	0.58	0.72	0.38
AUC	0.70	0.70	0.90	0.42

## Discussion

This study retrospectively analyzed the preoperative CT images of patients with HICH, conducted texture analysis, predicted intracranial hypertension according to the textural features obtained, and compared them with traditional morphological features. The results showed that texture features had better prediction ability than morphological features, and the prediction ability of textural features varied in different regions.

With the promotion of noninvasive concept, CT image-based ICP evaluation has been carried out. Clinically, it is commonly used to evaluate ICP based on visual features, including midline shift, width of third ventricle, change of basal cistern, and optic nerve sheath diameter. These radiological findings are regarded as morphological features and a prognostic evaluation system such as Marshall classification and Rotterdam classification has been formed, which is a concentrated manifestation of the clinical application of these features. Oliveira et al. (13) evaluated intracranial conditions by measuring the width of the third ventricle, peri-mesencephalic cistern, and sylvian fissure. Optic nerve sheath diameter had also been measured on preoperative brain CT, which predicted increased ICP with good discrimination and high inter-observer reliability (14). Rotterdam classification is a new CT scoring method proposed by Mass et al. (10) on the basis of Marshall classification, which was scored on the shape of basal cistern, midline shift, presence of the hematoma or contusion and presence of intraventricular hemorrhage or subarachnoid hemorrhage; the score indicated the severity of the brain injury (15). The above methods can predict ICP to a certain extent, but they are highly subjective, highly affected by the heterogeneity of the disease, and difficult to be accurately quantified (hematoma volume, midline shift, and ventricle cisternal size are currently measured by different methods and most of them are manually measured). In addition, most studies on morphological features were focused on individual correlation analysis, and comprehensive assessment models are rarely reported.

Currently, the main trend of texture analysis is to judge or predict the outcome of a clinical event through the extraction and deep learning of textural features of selected lesions. For example, a study extracted textural features to predict ICP level (elevated ICP with ICP > 12 mm Hg and normal ICP with ICP ≤ 12 mm Hg) in patients with traumatic brain injury (16). Shen et al. (17) filtered the CT images in different degree, and the gray related parameters (average gray intensity, variance, and uniformity) of hematoma sites on images with different texture thickness were extracted, so as to predict the possibility of early hematoma enlargement in patients with ICH. Texture features generally include first-order features, second-order features and higher-order features according to the current study of radiomics (18–20). CT value is the expression form of gray value on CT image, which describes distribution of the intensities of voxels within the ROI by entropy, mean value, range, root mean square, skewness, standard deviation, consistency, variance, and other parameters (known as first-order features). As mentioned above, CT value can be used to predict clinical events under certain conditions, but it has limitations for the assessment of ICP discussed in this study. For example, high CT value area caused by small amount of subarachnoid hemorrhage and low CT value area caused by brain edema existed around hematoma after ICH, both of which were under intracranial hypertension. These differences of CT value could cause effective visual impact, but they could not explain the same situation of intracranial hypertension caused by different CT values. Therefore, first order features were not included in this study.

The second-order features (known as textural features in a narrow sense) which described the spatial distribution of voxel intensity levels were obtained by GLCM and GLRLM. Guan et al. had showed the judgement ability of textural features. They selected 38 infarcted areas and 38 symmetrical non-infarcted areas of contralateral cerebral hemisphere from CT images by matching the lesion areas displayed on MRI images, extracted textural features, and established classifiers through computer deep learning method to verify its effectiveness. The results showed that there were differences in textural features between the two types of regions, which could be helpful for early clinical assessment of acute ischemic stroke and quantification of affected regions (21). However, the results of studies on the predictive ability of textural features were not always positive. Nawabi et al. (22) indicated that machine learning–based evaluation of filter- and texture-derived high-end image features provided the same discriminatory power in predicting functional outcome as multidimensional clinical scoring systems. In summary, most textural analysis based on second-order features at present focuses on selecting hematoma or the local lesion, while multi-area comparative analysis of surrounding tissues is rarely reported. This study aims to explore the differences in the degree of compression of local brain tissue after HICH by comparing the textural features of the brain tissue in bilateral symmetric regions without lesion, and to explore the relationship between these differences and ICP.

HICH is usually unilateral, and the main bleeding sites are basal ganglia, thalamus, ventricle, cerebellum and brain stem (23, 24). Patients with hemorrhage of ventricle, cerebellum, and brainstem were excluded in this study, so as to reflect the pressure changes in bilateral brain tissues caused by intracerebral hematoma. After the ICH, compression of hematoma and swelling of brain tissue result in the disruption of cranial cavity, such as midline deviation to the healthy side, ventricle compression, and decrease of VCR. Therefore, the average CT value of brain tissue on the bleeding side is higher than that on the opposite side. In clinical practice, doctors often judge the degree of increased ICP according the “density” of brain tissue, but it is greatly affected by anatomical factors and blood flow effects, and there are many application restrictions. The second-order textural features obtained by GLCM and GLRLM describe the relative positions of the two voxels. By comparing the textural features at the same position on the bleeding side and the contralateral side, the differences in the internal structure changes of the bilateral brain tissues after hematoma compression and brain tissue swelling can be reflected. In this study, variation rate was used to quantify the difference. The results showed that the three models of textural feature are superior to the MF model. The parameters included by the morphological model were commonly used in clinical practice to roughly evaluate intracranial hypertension, and their measurements were greatly affected by clinical experience, so the performance of the MF model is poor. In addition, the performance of PH model was better than that of AH model and TL model. The reason may lie in: the anterior horn and temporal lobe were close to the hematoma; due to the relative smaller space of the frontal lobe and temporal lobe compared with the occipital lobe, and the mass effect of hematoma, although the increase of hematoma would lead to the displacement of internal structures, the deformation space of the frontal or temporal brain tissue was limited, and was not as large as that of the occipital lobe. Therefore, the PH model has the best judgement ability.

In summary, the value and potential of textural analysis in the prediction of intracranial hypertension had been proved, but this study also had some limitations. Firstly, the sample size was relatively small and from a single center. Secondly, there are strict requirements on the inclusion data of texture analysis. In this study, hemorrhage of ventricle, cerebellum and brainstem were excluded, and the study coverage was narrow. Thirdly, as different observers have different perceptions of lesions in ROI delineation, manual segmentation would cause certain interference to experimental results, increase inaccuracy and lead to certain errors. Fourthly, the ICP value acquired from the intraventricular ICP sensor could not represent the actual ICP of different regions. Fifthly, this study was conducted only from the perspective of CT image; many objective factors including age, preoperative medication, and time of onset were not considered. Lastly, the HU method evaluated ICP values in a semiquantitative way, without providing a continuous value. These flaws need to be addressed in subsequent research.

## Conclusions

In summary, texture analysis, which is known for high-throughput, obtain a large amount of additional quantitative data that cannot be observed by eyes through parameterization of CT images, which can be used for the prediction and avoidance of adverse clinical events. In this study, compared with traditional morphological analysis, this novel method can distinguish intracranial hypertension more accurately, and has an extensive application prosperity for noninvasive filed.

## Data Availability Statement

The original contributions presented in the study are included in the article/Supplementary Material, further inquiries can be directed to the corresponding author/s.

## Ethics Statement

The studies involving human participants were reviewed and approved by the Ethics Committee of Shanghai General Hospital, Shanghai Jiao Tong University School of Medicine. The patients/participants provided their written informed consent to participate in this study.

## Author Contributions

GG, YS, YL, and XW conceived the study. GG and YX supervised and coordinated all aspects of the work. YS, YL, XW, and GZ collected and analyzed the data. YS, YL, XW, and GG wrote the paper. GG and YX acquired the funding and administrated the project. All authors contributed to the article and approved the submitted version.

## Funding

This study was supported by the National Natural Science Foundation of China (Grant Nos. 81671198 and 81971699) and the Clinical Research Innovation Plan of Shanghai General Hospital.

## Conflict of Interest

The authors declare that the research was conducted in the absence of any commercial or financial relationships that could be construed as a potential conflict of interest.

## Publisher's Note

All claims expressed in this article are solely those of the authors and do not necessarily represent those of their affiliated organizations, or those of the publisher, the editors and the reviewers. Any product that may be evaluated in this article, or claim that may be made by its manufacturer, is not guaranteed or endorsed by the publisher.
