# Supplementary material for: Evaluation of Intracranial Hypertension in Patients With Hypertensive Intracerebral Hemorrhage Using Texture Analysis
Source: Front Neurol. 2022 Mar 16;13:832234. doi: 10.3389/fneur.2022.832234 (PMC8966839; doi:10.3389/fneur.2022.832234)
Supplement: Supplementary file 2 [file Table_2.DOCX]

**1. Autocorrelation**

Autocorrelation is a measure of the magnitude of the fineness and coarseness of texture.

**2. Cluster Prominence**

Cluster Prominence is a measure of the skewness and asymmetry of the GLCM. A higher value implies more asymmetry about the mean while a lower value indicates a peak near the mean value and less variation about the mean.

**3. Cluster Shade**

Cluster Shade is a measure of the skewness and uniformity of the GLCM. A higher cluster shade implies greater asymmetry about the mean.

**4. Correlation**

Correlation is a value between 0 (uncorrelated) and 1 (perfectly correlated) showing the linear dependency of gray level values to their respective voxels in the GLCM.

**5. Gray Level Non-Uniformity (GLN)**

GLN measures the similarity of gray-level intensity values in the image, where a lower GLN value correlates with a greater similarity in intensity values.

**6. Gray Level Variance (GLV)**

GLV measures the variance in gray level intensity for the runs.

**7. High Gray Level Run Emphasis (HGLRE)**

HGLRE measures the distribution of the higher gray-level values, with a higher value indicating a greater concentration of high gray-level values in the image.

**8. Inverse Difference (ID)**

ID is another measure of the local homogeneity of an image. With more uniform gray levels, the denominator will remain low, resulting in a higher overall value.

**9. Inverse Difference Moment (IDM)**

IDM is a measure of the local homogeneity of an image. IDM weights are the inverse of the Contrast weights (decreasing exponentially from the diagonal i=j in the GLCM).

**10. Informational Measure of Correlation (IMC) 1**

IMC1 assesses the correlation between the probability distributions.

**11. Informational Measure of Correlation (IMC) 2**

IMC2 also assesses the correlation between the probability distributions.

**12. Joint Average**

Returns the mean gray level intensity of the distribution.

**13. Joint Energy**

Energy is a measure of homogeneous patterns in the image. A greater Energy implies that there are more instances of intensity value pairs in the image that neighbor each other at higher frequencies.

**14. Long Run Emphasis (LRE)**

LRE is a measure of the distribution of long run lengths, with a greater value indicative of longer run lengths and more coarse structural textures.

**15. Long Run High Gray Level Emphasis (LRHGLE)**

LRHGLRE measures the joint distribution of long run lengths with higher gray-level values.

**16. Maximum Probability**

Maximum Probability is occurrences of the most predominant pair of neighboring intensity values.

**17. Maximal Correlation Coefficient (MCC)**

The Maximal Correlation Coefficient is a measure of complexity of the texture and 0≤MCC≤10≤MCC≤1.

**18. Run Entropy (RE)**

RE measures the uncertainty/randomness in the distribution of run lengths and gray levels. A higher value indicates more heterogeneity in the texture patterns.

**19. Run Length Non-Uniformity (RLN)**

RLN measures the similarity of run lengths throughout the image, with a lower value indicating more homogeneity among run lengths in the image.

**20. Run Length Non-Uniformity Normalized (RLNN)**

RLNN measures the similarity of run lengths throughout the image, with a lower value indicating more homogeneity among run lengths in the image. This is the normalized version of the RLN formula.

**21. Run Percentage (RP)**

RP measures the coarseness of the texture by taking the ratio of number of runs and number of voxels in the ROI.

**22. Short Run Emphasis (SRE)**

SRE is a measure of the distribution of short run lengths, with a greater value indicative of shorter run lengths and more fine textural textures.

**23. Short Run Low Gray Level Emphasis (SRLGLE)**

SRLGLE measures the joint distribution of shorter run lengths with lower gray-level values.

**24. Sum Average**

Sum Average measures the relationship between occurrences of pairs with lower intensity values and occurrences of pairs with higher intensity values.

**25. Sum of Squares**

Sum of Squares or Variance is a measure in the distribution of neigboring intensity level pairs about the mean intensity level in the GLCM.
